# Cardiolipin Strongly Inhibits the Leakage Activity of the Short Antimicrobial Peptide ATRA-1 in Comparison to LL-37, in Model Membranes Mimicking the Lipid Composition of *Staphylococcus aureus*

**DOI:** 10.3390/membranes13030304

**Published:** 2023-03-06

**Authors:** Nathalia Calderón-Rivera, Jessica Múnera-Jaramillo, Sara Jaramillo-Berrio, Elizabeth Suesca, Marcela Manrique-Moreno, Chad Leidy

**Affiliations:** 1 Biophysics Group, Physics Department, Universidad de los Andes, Bogotá 111711, Cundinamarca, Colombia; 2 Faculty of Natural of Exact Sciences, Chemistry Institute, University of Antioquia, A.A. 1226, Medellin 050010, Antioquia, Colombia

**Keywords:** *Staphylococcus aureus*, antimicrobial peptides, lipid-peptide interaction, membrane active peptides, fluorescence spectroscopy, infrared spectroscopy

## Abstract

Cardiolipin is one of the main phospholipid components of *Staphylococcus aureus* membranes. This lipid is found at varying concentrations in the bilayer, depending on the growth stage of the bacteria, and as a response to environmental stress. Cardiolipin is an anionic phospholipid with four acyl chains, which modulates the bending properties of the membrane due to its inverted conical shape. It has been shown to inhibit the pore forming activity of several antimicrobial peptides, in general doubling the peptide concentration needed to induce leakage. Here we find that the short snake-derived antimicrobial peptide ATRA-1 is inhibited by several orders of magnitude in the presence of cardiolipin in saturated membranes (DMPG) compared to the human cathelicidin LL-37, which is only inhibited two-fold in its leakage-inducing concentration. The ATRA-1 is too short to span the membrane and its leakage activity is likely related to detergent-like alterations of bilayer structure. Fluorescence spectroscopy shows only a minor effect on ATRA-1 binding to DMPG membranes due to the presence of cardiolipin. However, FTIR spectroscopy shows that the acyl chain structure of DMPG membranes, containing cardiolipin, become more organized in the presence of ATRA-1, as reflected by an increase in the gel to liquid-crystalline phase transition temperature. Instead, a depression in the melting temperature is induced by ATRA-1 in DMPG in the absence of cardiolipin. In comparison, LL-37 induces a depression of the main phase transition of DMPG even in the presence of cardiolipin. These data suggest that cardiolipin inhibits the penetration of ATRA-1 into the membrane core, impeding its capacity to disrupt lipid packing.

## 1. Introduction

Cationic antimicrobial peptides (cAMPs) are small molecules between 10 and 50 amino acids in length, synthesized by a variety of organisms as a primary immune response against bacterial infections [1]. These peptides present a net positive charge, which results in a strong electrostatic interaction to microorganisms due to the abundance of anionic phospholipids in the outer leaflet of bacterial membranes. This leads to preferential binding of cAMPs to bacterial membranes, reducing the interaction with eukaryotic membranes [2,3]. One of the main modes of action of these antimicrobial peptides is the formation of pores in the bacterial membrane, inducing the dissipation of the bacterial ionic gradients, resulting in cell death [4]. In this mode of action, the peptides adhere on the membrane surface, and, after reaching a threshold surface concentration, insert into the membrane and aggregate leading to the formation of a polypeptide pore structures that can alters ion gradients. The insertion process requires overcoming a free energy barrier involving the translocation of the cationic peptide through the membrane hydrophobic core, which normally involves high levels of local curvature in the membrane. Other antimicrobial peptides, for example those that are too short to span the membrane, do not form polypeptide pore structures, but induce leakage by binding to the membrane in a detergent-like mechanism, altering membrane structure. This mode of action is referred to some times as the carpet model [5].

Gram-positive bacteria contain different anionic phospholipid species. Two of the most prevalent are phosphatidylglycerol (PG) and cardiolipin (CL) [6]. Cardiolipin is characterized by the presence of four acyl chains and two anionic charges. This unusual structure leads to an inverted conical shape that has been proposed to alter the bending properties and increase the mechanical strength of the membrane. Evidence to support this includes an increase in resistance to osmotic stress in cells with increased CL levels [7]. Cardiolipin has also been shown to increase the resistance to cationic antimicrobial peptide activity in mono-unsaturated acyl chain membranes [8], and other membrane active enzymes such as PLA_2_ [9]; however, it is still unclear how this inhibitory activity varies from peptide to peptide, and what are the underlying mechanisms for this inhibition.

In this work, we address this question by comparing the inhibitory activity of CL in model membranes composed of saturated acyl chains for two different antimicrobial peptides with significantly different lengths, the human cathelicidin LL-37 and the short motif ATRA-1 derived from Chinese cobra (*Naja atra*) cathelicidin. Saturated acyl chains are prevalent in Gram-positive bacteria such as the opportunistic pathogen such *Staphylococcus aureus* (*S. aureus*) and are therefore relevant in the study of antimicrobial activity. We address the underlying mechanism for this inhibitory effect by exploring changes in the binding affinity of these antimicrobial peptides in the presence of CL by fluorescence microscopy. We also explore the effect of increasing concentrations of cardiolipin on the mechanical strength of the membrane by subjecting liposomes to osmotic stress, showing that cardiolipin does indeed increase the mechanical strength of the membrane. Finally, Fourier Transform Infrared Spectroscopy (FTIR) experiments were performed to investigate how CL affects the interaction of LL-37 and ATRA-1 with the hydrophobic core of the lipid bilayer. This was done by exploring shifts in the gel to liquid-crystalline phase transition temperature of the model membranes.

## 2. Materials and Methods

### 2.1. Synthetic Lipid Systems and Peptide Structure Prediction

Based on the membrane lipid composition of *S. aureus*, the two major lipid components of the bacterial plasma membrane were chosen to obtain the synthetic lipid systems used in this study. According to the literature, *S. aureus* membranes are mainly composed of phosphatidylglycerol (80%) and CL (20%) [8]. The synthetic lipids used in the experiments were 1,2-dimyristoyl-sn-glycero-3-phospho-(1′-rac-glycerol) (DMPG, Lot. 140PG-167) and 1′,3′-bis [1,2-dimyristoyl-sn-glycero-3-phospho]-glycerol (TMCL, Lot. 750332P-200MG-A-030) from Avanti Polar Lipids (Alabaster, AL, USA).

Peptides LL-37 (LLGDFFRKSKEKIGKEFKRIVQRIKDFLRNLVPRTES, Lot. V1440EE070/PE1324) and ATRA-1 (KRFKKFFKKLK-NH_2_, Lot. V1440EE070-3/pe1316) were purchased, according to their reported sequences using the solid-phase method, from GenScript (Piscataway Township, NJ, USA). The purity of peptides was determined by analytical HPLC, and in all cases was higher than 95%. Their molecular weight was confirmed by MALDI-TOF mass spectrometry. All other reagents of analytical grade were obtained from Sigma–Aldrich. The helical wheel projections were obtained with the online tool NetWheels: Peptides Helical Wheel and Net projections maker (http://lbqp.unb.br/NetWheels, accessed on 10 December 2022). As the 3-Dimensional structure of ATRA-1 peptide is unknown, de novo peptide structure prediction package PEP-FOLD3 was used to predict the structure (http://bioserv.rpbs.univ-paris-diderot.fr/, accessed on 10 December 2022) [10,11,12].

### 2.2. Fluorescence Spectroscopy

Four lipid systems were prepared for the calcein leakage experiments with different DMPG:CL molar ratios (100:0, 95:5, 90:10 and 80:20). An appropriate amount of the lipids (DMPG and CL) was dissolved using chloroform in glass test tubes to reach a concentration of 10 mM. The solvent was evaporated under a stream of nitrogen, and the samples were then kept under vacuum for at least 12 h to ensure complete solvent removal. The lipid films were hydrated and resuspended in buffer (20 mM HEPES, 125 mM NaCl at pH 7.4) containing 50 mM calcein (400 mOsm). Test tubes were vortexed for 5 min to obtain multilamellar vesicles (MLVs). The calcein buffer was prepared as follows: 778.2 mg of calcein was dissolved in 5 mL of 1 M NaOH. After the calcein was fully dissolved, 2.5 mL of 10× HEPES solution (100 mM HEPES) was added together with 5 mL of MilliQ water (Millipore, Bedford, MA, USA). Volume was completed dropwise with HCl in order to reach pH 7.4. An appropriate amount of NaCl solution was then added to reach a concentration of 400 mOsM. Finally, MilliQ water was then added to bring the calcein buffer to a final volume of 25 mL and a final calcein concentration of 50 mM. The sample was warmed to 60 °C for 5 min and vortexed for 1 min, this process was repeated five times. Large Unilamellar Vesicles (LUVs) were obtained by extruding the suspension 21 times through two polycarbonate filters of 100 nm pore size, with the use of a mini-extruder from Avanti Polar Lipids (Alabaster, AL, USA). The dye-containing vesicles were separated from non-entrapped calcein using a size exclusion chromatographic column Sephadex G50 (fine, 10 mm × 250 mm) eluted with HEPES buffer that did not contain calcein. As DMPG has a gel to liquid-crystalline phase transition temperature at around 24 °C the separation process was conducted at a temperature at least 10 °C above the phase transition temperature by placing the column inside an incubator 30 min before separation and during the separation process. The collected samples were always kept warm before measurements were conducted. The fluorometer cuvette with buffer was always stabilized to a final temperature of 37 °C before adding the vesicles.

For Laurdan generalized polarization (GP) measurements on LUVs, the dye was added to lipids in chloroform to a concentration of 1:1000 (probe to lipid ratio) and the calcein buffer was replaced by the elution buffer. Fluorescence measurements were monitored using a PC-1 ISS spectrofluorometer (Urbana, IL, USA) equipped with a temperature controller. Slides and grids were selected depending on sample intensity and photobleaching sensitivity. For calcein release experiments, excitation and emission wavelengths were set at 490 and 520 nm, respectively. For Laurdan GP experiments, the sample was excited at 350 nm and the emission was recorded at 440 and 500 nm. All measurements were carried out at 37 °C using 1 mL of sample in a cuvette with a final concentration of 30 μM of lipid, and sample homogeneity was maintained through continuous magnetic stirring.

Calcein leakage caused by loss of membrane integrity was induced varying the concentration of peptide in the sample or, in the case of the osmotic stress experiments in the absence of peptides, by varying the ratio dH_2_O/buffer in the cuvette. The complete release of calcein was achieved through the addition of 1% volume Triton-X. The apparent percentage of calcein release (normalized membrane leakage), was calculated according to Equation (1):

(1) $$L_{T}=\frac{F-F_{0}}{F_{100}-F_{0}}\times 100$$

For Laurdan generalized polarization measurements, liposomes were incubated with peptides for 5 min allowing the sample to stabilize. Emission spectra were collected and generalized polarization was calculated in the standard manner by finding the averaged difference in intensities at 440 nm and 490 nm as shown in Equation (2):

(2) $$GP=\frac{I_{440}-I_{500}}{I_{440}+I_{500}}$$

Membrane tension was calculated by first obtaining the osmotic pressure gradient using the Van ’t Hoff equation:

$$\Delta p=\Delta ck_{B}T$$

where Δp is the osmotic pressure differential across the membrane, Δc is the osmolarity difference between the inside and the outside of the membrane, $k_{B}$ is Boltzmann’s constant, and *T* is the temperature in Kelvin. From the pressure gradient, the membrane lateral surface tension Σ can be estimated using the Laplace equation.

$$\Sigma =\frac{R\Delta p}{2}$$

where R is the average radius of the liposome, which we estimate to be 50 nm.

### 2.3. Fourier Transform Infrared Spectroscopy

Solid supported lipid bilayers were prepared in situ on a BioATR II cell. The unit was integrated to a Tensor II spectrometer (Bruker Optics, Ettlingen, Germany) with a liquid nitrogen MCT detector using a spectral resolution of 4 cm^−1^ and 120 scans per spectrum. The desired temperature was set by a computer-controlled circulating water bath Huber Ministat 125 (Huber, Offenburg, Germany). First, the background was taken using HEPES buffer 20 mM, 500 mM NaCl and 1 mM EDTA in the same temperature range. Subsequently, for coating the silicon crystal, stock solutions of the lipids were dissolved in chloroform. The cell was filled with 20 µL of a 20 mM lipid stock solution (DMPG, CL or DMPG:CL (80:20)), and the chloroform was evaporated resulting in a lipid multilayer film. For in situ measurements, the cell was afterwards filled with 20 µL of buffer or peptide solution and incubated over the phase transition temperature for 10 min. To determine the position of the vibrational band in the range of the second derivative of the spectra, all the absorbance spectra were cut in the 2970–2820 cm^−1^ range, shifted to a zero baseline and the peak picking function included in OPUS software was used. The results were plotted as a function of the temperature. To determine the transition temperature (T_m_) of the lipids, the curve was fitted according to the Boltzmann model to calculate the inflection point of the obtained thermal transition curves using the OriginPro 8.0 software (OriginLab Corporation, Northampton, MA, USA).

## 3. Results

### 3.1. Prediction of the 3D Structure of LL-37 and ATRA-1

The physicochemical characteristics of the peptides LL-37 and ATRA-1 are summarized in Table 1. The cathelicidin peptide LL-37 is of human origin with broad antimicrobial activity towards Gram-positive and Gram-negative bacteria, which forms part of the primary immune response in humans [13,14,15,16]. This widely studied antimicrobial peptide is 37 residues in length and presents an amphipathic α-helical structure, both in aqueous solutions and when bound to bilayer membranes [17,18]. The LL-37 initially binds parallel to the membrane, and, after reaching a threshold concentration, inserts vertically. Different insertion and aggregation mechanisms have been proposed that appear to be highly sensitive to the peptide to lipid ratio, and its ability to stabilize the inverted hexagonal phase has been used as evidence of a toroidal pore structure during pore formation, but only for certain concentrations [19].

**Figure 1:**
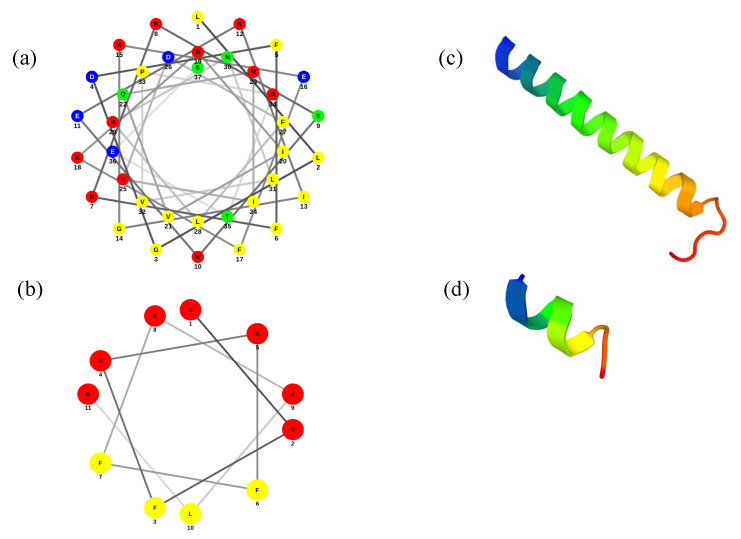
Prediction of helical structure for (**a**) LL-37 and (**b**) ATRA-1. Polar residues are represented as: basic in red, acid in blue, and uncharged in green: Non-polar residues are represented in yellow. Three-dimensional structures of (**c**) LL-37 and (**d**) ATRA-1.

The antimicrobial peptide ATRA-1 is derived from the Chinese cobra (*Naja atra*) cathelicidin (NA-CATH), showing similar antimicrobial activity compared to the original snake Cathelicidin [20,21]. The ATRA-1 is highly cationic, with a total charge of +8 and only 11 residues in length. Although it has been proposed to act through membrane disruption, its mode of action has not yet been elucidated. Its short length would prevent the peptide from forming pores through aggregation of vertically inserted peptides, suggesting a mechanism more closely related to a detergent-like carpet model. With the purpose of studying the differences between both peptides, the helical wheel projections and the secondary structures were obtained using the online PEP-FOLD3 package. Figure 1 presents the prediction of the α-helical secondary structure of ATRA-1 and LL-37, showing an amphipathic structure with two faces for both peptides, where one face is enriched in hydrophobic residues and the other forming a polar cationic surface.

According to the PEP-FOLD3 results, the prediction of the secondary structure of ATRA-1 shows a short α-helix (Figure 1c), while the prediction of LL-37 shows an α-helical structure with the N-terminal region less perfectly formed. These results are in accordance with previous reports [17]. The most significant difference between the two peptides is their relative length, where LL-37 can span the membrane when inserted while ATRA-1 does not. With a pitch length of 5.4 Å per turn for an alpha helix, LL-37 would have a total length, as an alpha helix structure, of approximately 40 Å. This matches the average width of a bilayer membrane. In contrast, ATRA-1 would only measure approximately 11 Å.

### 3.2. Pore Forming Activity of LL-37 and ATRA-1 in Model Membrane Systems

**Figure 2:**
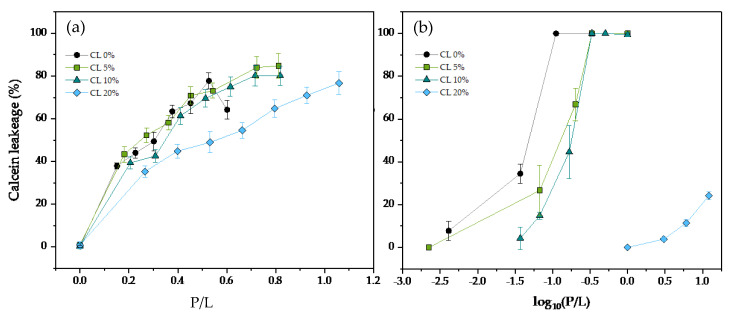
Percentage of calcein release as a function of the peptide to lipid molar ratio (P/L) for (**a**) LL−37, and (**b**) ATRA−1 in DMPG 100 nm unilamellar vesicles containing varying molar percentages of CL. All measurements were performed at 37 °C.

Calcein leakage experiments were conducted to measure antimicrobial peptide activity in DMPG liposomes containing varying amounts of the cardiolipin CL at 37 °C. Both phospholipids have 14-carbon saturated acyl chains, mimicking the bacterial membrane composition, since saturated chains are predominant in Gram-positive bacteria such as *S. aureus*. In Figure 2, we tested the calcein leakage assay for the human cathelicidin LL-37 and the snake-derived peptide ATRA-1 respectively. In the case of LL-37 (Figure 2a), there is a gradual increase in the protein to lipid (P/L) ratio needed to induce leakage, approximately doubling the amount of LL-37 needed to induce 50% leakage at a CL content of 20 mol%. For ATRA-1 (Figure 2b), the inhibition levels are stronger, with an increase of more than two-orders of magnitude in the P/L needed for the onset of leakage.

**Figure 3:**
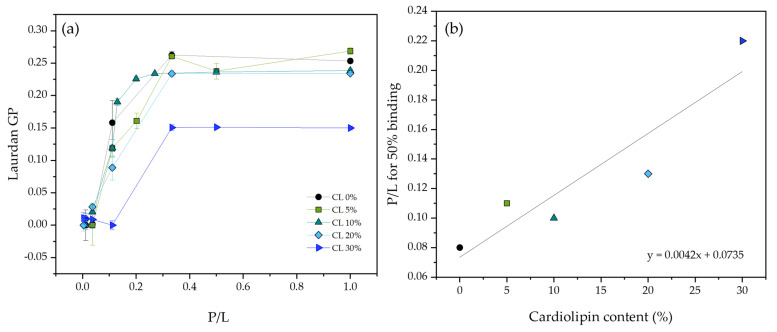
(**a**) ATRA−1 binding isotherms in the presence of DMPG unilamellar vesicles containing varying amounts of CL, as measured by monitoring Laurdan generalized polarization. (**b**) P/L ratio needed to reach 50% binding is plotted as a function of cardiolipin content. All measurements were performed at 37 °C.

To investigate if changes in the binding affinity to the membrane could explain the reduction in ATRA-1 activity in the presence of CL, binding isotherms were obtained by monitoring the Laurdan GP signal as a function of the peptide to lipid ratio for varying amounts of ATRA-1 at different compositions (Figure 3). The fluorescence emission spectrum of Laurdan is sensitive to protein binding, providing a tool to measure the binding isotherms. Laurdan positions itself near the membrane headgroup/acyl-chain interface and has been shown to be sensitive to the surrounding polar environment. In particular, because the liquid-crystalline to gel phase transition is characterized by a sharp change in the amount of water at the interface, Laurdan is often used to study lipid melting behavior. As peptide attachment to the membrane will change the polar environment of the surface, we take advantage of this sensitivity to monitor peptide binding. Binding will lead to a shift in Laurdan GP that should be approximately proportional to the bound peptide. Specifically, how the peptide is affecting Laurdan GP as it binds goes beyond the scope of this paper. However, it suffices to know that Laurdan GP is sensitive to this binding event. The results show that the P/L ratio to reach 50% binding approximately doubles in the presence of 20 mol% cardiolipin, indicating that ATRA-1 binding affinity to DMPG membranes is not strongly affected by the presence of cardiolipin. More thorough experiments are needed to be able to report accurately a peptide to membrane binding constant. However, the binding isotherms that we report are sufficient to show that binding is not greatly affected by the presence of CL.

**Figure 4:**
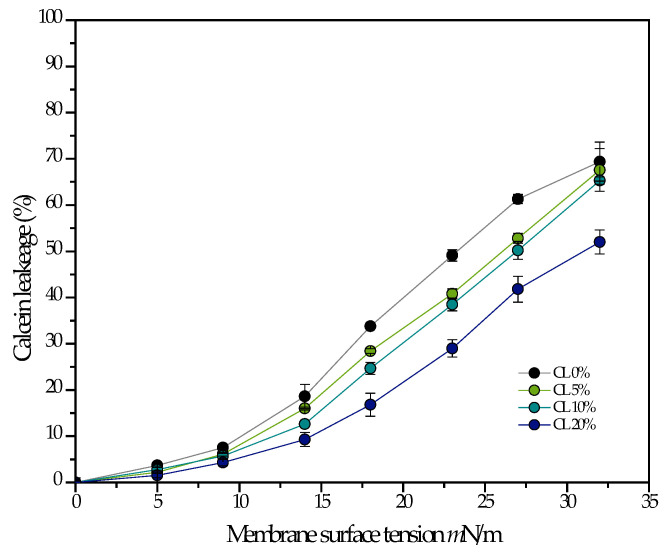
The percentage of leakage is measured as a function of membrane tension induced by osmotic stress in 100 nm liposomes composed of pure DMPG, and DMPG with varying amounts of CL. All measurements were performed at 37 °C.

Cardiolipin has been proposed to confer mechanical resistance to bacterial membranes. In particular, bacteria overproduce cardiolipin when exposed to high salinity or osmotic stress [22]. For this reason, an experiment that would show this behavior in a model system was performed by fluorescence spectroscopy. In Figure 4, calcein containing DMPG liposomes with varying amounts of cardiolipin were subjected to osmotic stress, and the percentage of content leakage was quantified. The results show that the percentage of leakage increases monotonically as a function of membrane tension, but to a lesser extent when the liposomes contain cardiolipin, supporting the notion that cardiolipin does increase the mechanical strength of the membrane, at least to tension induced pore formation.

### 3.3. FTIR Experiments on the Interaction of Antimicrobial Peptides with Model Membranes

Chemical bonds undergo different forms of vibrations such as stretching, twisting, and rotating, when energy is absorbed. Infrared spectroscopy (IR) measures these vibrations and provides information regarding molecular structure and structural interactions of biomolecules in different environments, such as in water, thin films, organic solvents, detergent micelles as well as in lipid bilayer matrix [23]. Biological membranes are primarily composed of phospholipids. The FTIR is well suited to follow thermotropic lipid phase transitions, which occur between the lamellar gel and liquid-crystalline states when the lipids contain in their acyl chains all *trans* and *gauche* rotamers, respectively. The transition is a reorganization of the system’s components in response to changes in the free energy of the system. The FTIR spectroscopy allows the study of motional freedom of CH_2_ absorbing groups within the acyl chains of phospholipid molecules and the changes due to temperature, pH, solutes, and the interaction with exogenous molecules like peptides [24]. The FTIR spectra of hydrated samples of several phospholipids can be measured in the absence and presence of a peptide as a function of temperature and at different lipid:peptide ratios. From the temperature dependence of the wavenumber of the CH_2_ symmetric stretching mode, it is possible to calculate the gel to liquid-crystalline phase transition temperature (T_m_). The FTIR spectroscopy has also been used to study the effect of hydration on phospholipids [25]. To follow the conformational order of the lipid chains in the *S. aureus* model membranes under study, we monitored the symmetric CH_2_ vibrational mode (ν_s_CH_2_) in the spectral range of 2970 to 2820 cm^−1^, as an indicator for the gel-to-fluid membrane phase transition. The increasing temperature-induced changes in the *trans–gauche* ratio of the acyl chains, and a shift in the maximum wavenumber position of this specific band is considered as a marker for the fluidity and change in phase behavior of the membrane. In the gel phase, ν_s_CH_2_ lies at 2850 cm^−1^ and in the liquid crystalline phase, around 2852 to 2853 cm^−1^ [26].

The phase transition results of the individual lipids showed an inflection point that corresponds to the main transition temperature (T_m_) from the gel to the liquid crystalline phase (L_β_–L_α_). The T_m_ observed for the pure DMPG and CL were 22.9 and 43.2 °C, respectively. These results are in accordance with previous reports of literature [27,28,29]. The mixture DMPG:CL showed a wider phase transition, characteristic of a mixture of lipids, with a T_m_ of 28.7 °C (Table 2).

**Figure 5:**
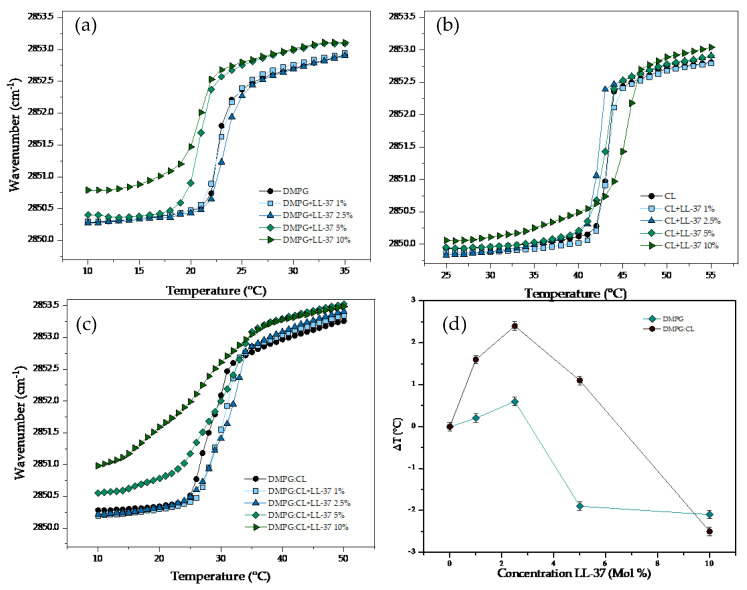
Peak positions of the symmetric stretching vibration bands of the methylene groups in dependence on temperature. Different concentrations of LL−37 in supported lipid bilayers of (**a**) DMPG, (**b**) CL and (**c**) DMPG:CL. (**d**) Change of T_m_ (△T) in the DMPG and DMPG:CL.

The results of the temperature dependence of the wavenumber values of the peak position of the interaction of LL-37 with supported bilayers of DMPG, CL and DMPG:CL are summarized in Figure 5. The interaction of the cathelicidin with the DMPG resulted in fluidization of the lipid system as well as a decrease of the T_m_ (Figure 5a). At the lowest concentrations evaluated, there was no significant effect of the peptide. However, at the concentrations of 5 and 10% molar percentage, a fluidization effect was detected when an increase in the wavenumber of the stretching ν_s_CH_2_ band was observed below and above the T_m_. The interaction of LL-37 with CL bilayers had a completely different effect, the peptide did not induce fluidization of the system (Figure 5b). Only at the highest concentration evaluated of LL-37 (10%), there was a slight fluidization effect in the gel phase with an upward shift of T_m_ (45.1 °C). The results of the interaction of LL-37 with the DMPG:CL mixture is presented in Figure 5c. At the lowest concentrations evaluated (1 and 2.5%), the peptide induced an increase of the T_m_ up to 31.1 °C. However, increasing concentrations induced a strong fluidization effect detected in the gel phase, and a shift downwards up to 2.5 °C of the main transition temperature. Figure 5d shows the variation in the main phase transition temperature (∆T) for DMPG and DMPG:CL lipid models in the presence of increasing LL-37 concentration. The results in Figure 5d show that LL-37 induces a change in the main phase transition for pure DMPG membranes at concentrations higher than 5%. For the DMPG:CL mixtures, the phase transition temperature increases at low concentrations of LL-37, but decreases at higher LL-37 concentrations, indicating an insertion of the peptide.

**Figure 6:**
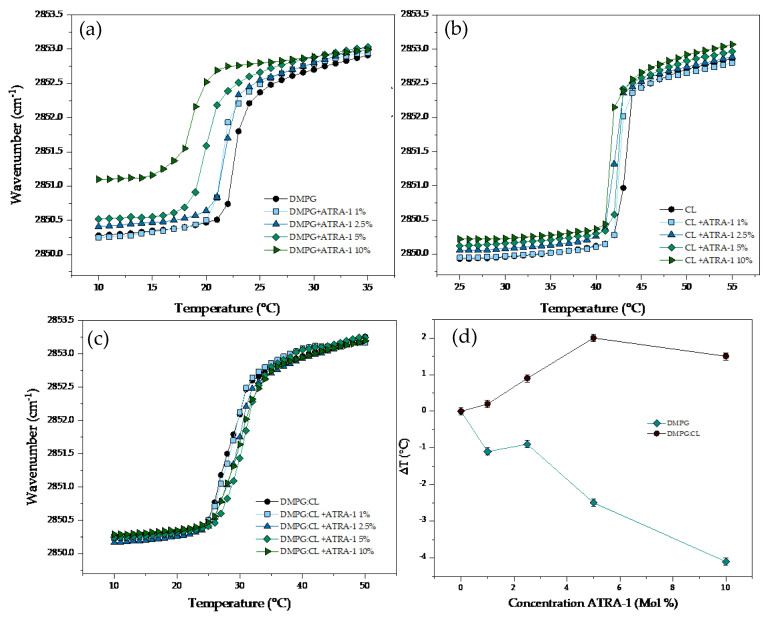
Peak positions of the symmetric stretching vibration bands of the methylene groups as a function of temperature. Different concentrations of ATRA−1 in supported lipid bilayers of (**a**) DMPG, (**b**) CL and (**c**) DMPG:CL. (**d**) Change of T_m_ (△T) in the DMPG and DMPG:CL.

The results of the temperature dependence of the wavenumber values of the peak positions of the DMPG, CL, and DMPG:CL acyl chains in the presence of ATRA-1 are presented in Figure 6. The interaction of ATRA-1 with DMPG lipid bilayers induced a strong fluidization effect even at the lowest concentration evaluated (1%) with a reduction of the T_m_ up to 1.1 °C (Figure 6a). Additional increasing concentrations of ATRA-1 induced progressive fluidization of the DMPG bilayers. The effect is evident in the gel phase, where the increase in the wavenumber was up to 1 cm^−1^ at a fixed temperature, and at the highest concentration evaluated of the peptide (10%), the T_m_ was shifted downwards by much as ca. 4.1 °C. The effect of the incubation of the peptide ATRA-1 with the CL bilayers is summarized in (Figure 6b). The interaction of the peptide induced a slight destabilization effect in the lipid bilayers in comparison with the effect observed in DMPG. Below and above the T_m_, increasing concentrations of ATRA-1 induced a slight increase in the fluidity, the effect is related with a mild reduction of the T_m_. All the results of the T_m_ are summarized in Table 3. The results of the interaction of ATRA-1 with DMPG:CL are presented in Figure 6c. Increasing concentrations of the peptide induced an opposite effect in comparison with the pure lipids. The incubation of the mixture of lipids with ATRA-1, induced a reduction in the fluidity of the lipid systems, with a gradual increase of the T_m_. At the highest concentration evaluated for ATRA-1 (10%), the temperature increases up to ca. 3.6 °C. Figure 6d shows the variation in the main phase transition temperature (∆T) for DMPG and DMPG:CL lipid models in the presence of increasing ATRA-1 concentration. The results in Figure 5d show that ATRA-1 induces a depression of the main phase transition for pure DMPG membranes at all peptide concentrations. For the DMPG:CL mixtures, the phase transition temperature increases and remains high at all the peptide concentrations tested.

In the presence of cardiolipin, the phase transition temperature increases at low concentrations of ATRA-1 and drops in temperature at higher ATRA-1 concentrations, indicating an insertion of the peptide.

## 4. Discussion

Cardiolipin is a major phospholipid component in bacterial membranes, with levels of CL in *S. aureus* reaching around 20 mol% of phospholipid content in the stationary phase [8]. The concentration of CL varies widely in *S. aureus* in response to environmental stress, such as high salinity or acidity [22,30], and changes drastically depending on the growth stage [31,32,33]. For this reason, it becomes relevant to understand the role of CL in regulating the activity of antimicrobial peptides to have a better picture of the effectiveness of these antimicrobial agents under different bacterial conditions. For example, there is evidence that the production of CL, triggered by human serum, induces resistance in *S. aureus* towards the clinically approved antimicrobial peptide daptomycin [34].

In a previous publication, a reduction in the antimicrobial lytic activity was observed for two different peptides (LL-37 and ΔM2) in the presence of 20 mol% CL in unsaturated model membranes. It was found that the concentration needed to reach 50% leakage doubled in the presence of CL [8]. A recent molecular dynamics study showed that there is an increase in the free-energy cost of forming pores in saturated membranes (DMPG) in the liquid-crystalline state when the membranes contain CL. This increase in energy cost was attributed to the negative curvature of the molecule which augmented the curvature energy cost of pore formation by participating directly in the pore structure [35]. The inhibition of antimicrobial peptides because of the negative curvature of CL has also been discussed extensively elsewhere [36,37,38]. Additionally, the increase in the free energy cost of pore formation is corroborated in the current study by the observed increased resistance to lysis by osmotic stress for DMPG membranes that contain CL (Figure 4).

While the inhibitory capacity of CL towards antimicrobial peptide pore-forming activity is evident, the current results show that there are strong differences in the levels of antimicrobial peptide inhibition by this lipid. While, for LL-37, the concentration to induce membrane leakage in DMPG doubles in the presence of 20 mol% CL, the concentration needed to induce leakage for the short antimicrobial peptide ATRA-1 increases by several orders of magnitude for the same system in the presence of 20 mol% CL. In the absence of CL, ATRA-1 is around two orders of magnitude more potent than LL-37 in DMPG membranes in the liquid-crystalline phase. In the presence of CL, the concentration of ATRA-1 needed to induce leakage increases to levels above those observed for LL-37.

Figure 3 shows that binding of ATRA-1 is only slightly affected by the presence of CL. This discards the possibility of a reduction in the binding affinity between the peptide and the membrane as the reason for the drastic reduction in ATRA-1 activity. With regards to the mechanical properties of the membrane, Figure 4 shows a linear increase in the mechanical resistance of the membrane to lysis in the presence of CL. This is likely to increase the energy costs of pore formation. However, it is not clear how this could affect ATRA-1 more drastically than LL-37.

Antimicrobial peptides, such as ATRA-1, that are too short to span the lipid bilayer, are thought to disrupt the membrane through a detergent-like mechanism known as the carpet model [39]. In this proposed mechanism, the peptides accumulate on the membrane surface and disrupt membrane structure, most likely by inducing positive intrinsic curvature in the lipid bilayer, leading to micellization of the membrane. CL is likely to interfere with this process. Podger et al. showed, through molecular dynamics simulations, that the short antimicrobial peptide Aurine 1.2 was unable to disrupt the lipid bilayer in the presence of CL, even at high surface coverage levels [36]. The peptide accumulated and induced membrane deformation but did not insert or disrupt membrane structure.

**Figure 7:**
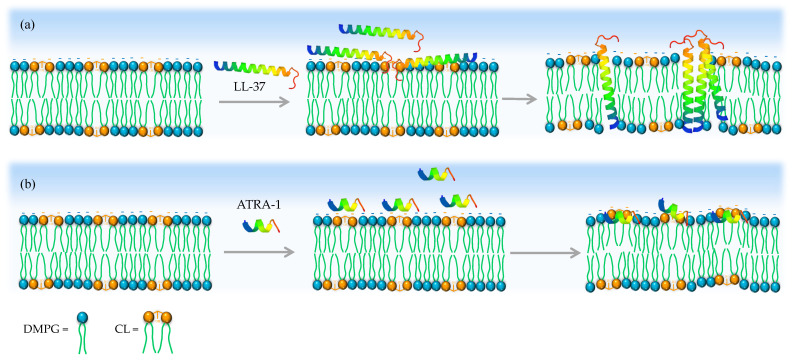
Proposed model for how CL inhibits ATRA-1 pore forming activity. (**a**) LL-37 can insert in the membrane after binding in the presence of CL. (**b**) In contrast, ATRA-1 binds to the membrane surface but does not insert into the membrane core in the presence of CL.

Turning our attention to ATRA-1, we observe by FTIR that both LL-37 and ATRA-1 depress the liquid–crystalline to gel phase transition temperature in pure DMPG membranes (Figure 5 and Figure 6). In the presence of CL, only LL-37 induces a depression of the phase transition at higher concentrations. In contrast, ATRA-1 leads to higher lipid packing levels at all peptide concentrations tested in the presence of CL, as indicated by an increase in the phase transition temperature. The FTIR data indicate that ATRA-1 binds to the membrane surface but does not insert, as a depression in the main phase transition temperature would be expected if the peptide inserted [40,41,42]. Instead, ATRA-1 binding drives a condensation of the lipids in the presence of CL (Figure 7). The positioning and accumulation of ATRA-1 on the membrane surface in the presence of CL is consistent with the results presented by Podger et al. for aurein 1.2 [36].

Cardiolipin contains two negatively charged (at pH 7) phosphate groups that could each generate electrostatic interactions with cationic amino acids in ATRA-1. This may play a role in stabilizing the peptide on the surface. In combination with the negative curvature of CL, these two factors may be enough to prevent insertion. It is interesting to note that the human cathelicidin LL-37, which forms part of the primary immune response in humans, is much less sensitive to the presence of CL. This could help maintain its effectiveness under different bacterial physiological conditions that induce variations in CL content.

## Figures and Tables

**Table 1:** Overview of the characteristics of the AMPs used in this study.

Peptide	Sequence	Charge	Hydrophobicity (%)
LL-37	LLGDFFRKSKEKIGKEFKRIVQRIKDFLRNLVPRTES	+6	37.8
ATRA-1	KRFKKFFKKLK-NH_2_	+8	36.4

**Table 2:** Phase transition temperatures (T_m_) of the three lipids systems in the presence of different concentrations of LL-37, as determined by FT-IR. Standard deviations are <0.1 °C.

LL-37 (mol%)		T_m_ (°C)	
	DMPG	CL	DMPG:CL
0	22.9	43.2	28.7
1	23.1	43.2	30.3
2.5	23.5	42.1	31.1
5	21.0	42.8	29.8
10	20.8	45.1	26.2

**Table 3:** Phase transition temperatures (T_m_) of the three lipids systems in the presence of different concentrations of ATRA-1, as determined by FT-IR. Standard deviations are <0.1 °C.

ATRA-1 (mol%)		T_m_ (°C)	
	DMPG	CL	DMPG:CL
0	22.9	43.2	28.7
1	21.8	42.6	28.9
2.5	22.0	42.0	29.6
5	20.4	42.4	30.7
10	18.8	41.7	30.2

## Data Availability

The data involved in this paper have been presented in articles and supporting materials in the form of diagrams or tables.
